# Sinusoidal Optic Flow Perturbations Reduce Transient but Not Continuous Postural Stability: A Virtual Reality-Based Study

**DOI:** 10.3389/fphys.2022.803185

**Published:** 2022-05-18

**Authors:** Jakob Ketterer, Steffen Ringhof, Dominic Gehring, Albert Gollhofer

**Affiliations:** Department of Sport and Sport Science, University of Freiburg, Freiburg, Germany

**Keywords:** postural control, virtual reality, sensory reweighting, sensory conflicts, optic flow perturbation

## Abstract

Optic flow perturbations induced by virtual reality (VR) are increasingly used in the rehabilitation of postural control and gait. Here, VR offers the possibility to decouple the visual from the somatosensory and vestibular system. By this means, it enables training under conflicting sensorimotor stimulation that creates additional demands on sensory reweighting and balance control. Even though current VR-interventions still lack a well-defined standardized metric to generate optic flow perturbations that can challenge balance in a repeatable manner, continuous oscillations of the VR are typically used as a rehabilitation tool. We therefore investigated if continuous sensory conflicts induced by optic flow perturbations can challenge the postural system sustainably. Eighteen young adults (m = 8, f = 10, age = 24.1 ± 2.0 yrs) were recruited for the study. The VR was provided using a state-of-the-art head-mounted display including the virtual replica of the real environment. After familiarization in quiet stance without and with VR, bipedal balance was perturbed by sinusoidal rotations of the visual scenery in the sagittal plane with an amplitude of 8° and a frequency of 0.2 Hz. Postural stability was quantified by mean center of mass speed derived from 3D-kinematics. A rmANOVA found increased postural instability only during the first perturbation cycle, i.e., the first 5 s. Succeeding the first perturbation cycle, visual afferents were downregulated to reduce the destabilizing influence of the sensory conflicts. In essence, only the transient beginning of sinusoidal oscillation alters balance compared to quiet standing. Therefore, continuous sinusoidal optic flow perturbations appear to be not suitable for balance training as they cannot trigger persisting sensory conflicts and hence challenge the postural system sustainably. Our study provides rationale for using unexpected and discrete optic flow perturbation paradigms to induce sustainable sensory conflicts.

## Introduction

To inhabit the world with all its unpredictable, variable environmental and situational contexts, a powerful yet flexible postural system is crucial (Horak, 2009). This flexibility of the postural system is guaranteed by appropriate changes in muscle activation that generate joint torques correcting for deviations from the desired orientation (Peterka, 2002). To orchestrate those adjustments, particularly in response to balance challenges, the central nervous system (CNS) requires reliable sensory feedback to generate efferent commands that produce corrective muscle torque to stabilize the human body. For this purpose, multiple sensory channels are simultaneously integrated in the CNS, including visual, vestibular, and somatosensory input. It has been demonstrated that the integration of sensory information appears to be dynamically regulated to adapt to changing environmental conditions and available sensory information (Hwang et al., 2014). Hereby, reliable sensory information from one sensory system is preferred over less reliable information from another sensory system (Kiemel et al., 2002). This is referred to as sensory reweighting (Nashner and Berthoz, 1978).

To improve the postural control system, whether to prevent falls in old age, to regain performance after injury, or for training purposes in sports, balance training is recommended to perturb the different sensory systems required for balance. Mostly, unstable support surfaces are used for this purpose (Taube et al., 2008), which force the subject to utilize the optimal source of sensory information. Closing the eyes or pitching the head (Johnson and Van Emmerik, 2012) can increase the difficulty of the balance task by further modifying the reliability of the visual and/or vestibular system, thus creating sensory conditions that are more challenging. The ability to select and reweight sensory information adaptively is considered one of the most important factors for postural stability, e.g. in the elderly (Horak et al., 1989b). Balance exercises that challenge the sensory systems and specifically target multisensory integration mechanisms were shown to improve sensory reweighting and balance control (Allison et al., 2018).

To increase the variability of balance exercises and provide broader access to sensory perturbations, virtual reality (VR) offers completely new possibilities (Chander et al., 2019). VR provides an interface between humans and computer systems that enables natural and intuitive interaction within the simulated three-dimensional environment, thereby allowing researchers to systematically modulate the visual input almost without limitations and by this means to manipulate the interaction between the organism and the environment in an arbitrary but still standardized way (Hedges et al., 2011). Whereas the visual input in conventional balance training usually is binary (eyes closed or eyes open), VR applications have the ability to decouple the visual from the somatosensory and vestibular systems in a more fine-grained manner by providing manifold possibilities of optic flow perturbations (Canning et al., 2020). By this means, it can induce conflicting sensorimotor stimulation that creates additional demands on sensory reweighting and balance control (Martelli et al., 2019) that are necessary to evoke cortical reorganization and neuroplasticity (Adamovich et al., 2009).

For instance, oscillating visual fields, i.e. moving room paradigms, can trigger these conflicts and the associated postural instability. The visual field in the virtual environment can be spatially manipulated to target the neuromuscular skills required for balance (Osaba et al., 2020). Adamovich et al. (2009) describe the incorporation of this element in balance training as the logical “next step”, as it may open a new direction in balance training and yields valuable implications to prevent falls or (sports-) injuries. In this context, Allison et al. (2018) examined the effect of sensory-challenge balance exercises on sensory reweighting capability in older adults. The authors found significant improvement in sensory reweighting and balance following balance exercises specifically targeting multisensory integration mechanisms through computerized, variable surface and/or visual environment motion. They concluded that their results provide a scientific rationale for sensory-challenge exercises to reduce fall risk. Based upon such findings, improvement in multisensory interactions has been suggested as a potentially fruitful area for new interventions (Bugnariu and Fung, 2007).

Consequently, the use of VR as a rehabilitation tool has advanced substantially within the last decade (Juras et al., 2019). There is growing evidence that when combined with conventional rehabilitation, VR offers improved benefits for balance and gait rehabilitation in neurological patients (for review, see Cano Porras et al., 2018). However, despite its growing popularity and proven efficacy to perturb balance and induce postural instability (Horlings et al., 2009; Chiarovano et al., 2017), information about optimal intervention programs (e.g., dosage and tasks) and defined paradigms for disturbing balance is still scarce. This hampers the development of both standardized interventions and the optimal use of VR in balance rehabilitation and balance training. Specifically, no established metric exists for creating a virtual environment that can perturb balance in an effective and repeatable manner. To be useful in balance training, however, the perturbation effects have to be well preserved in order to provide a permanent challenge to the postural system (Heidner et al., 2020).

Therefore, the purpose of this study was to investigate if continuous sensory conflicts can challenge the postural system sustainably and can thus deliver paradigms with additional demands on sensory reweighting and balance control processes. To provide a paradigm with conflicting sensorimotor stimulation, we used VR to create a synthetic replica of our real laboratory and to generate continuous rotatory oscillations of this virtual laboratory in the sagittal plane.

## Materials and Methods

### Subjects

Eighteen young adults (m = 8, f = 10, age = 24.1 ± 2.0 yrs) with no conditions affecting balance were recruited for this study. All volunteers had no previous experience regarding VR. The study was approved by the ethics committee of the University of Freiburg and in accordance with the declaration of Helsinki. The participants provided their written informed consent to participate in this study.

### Virtual Environment

**FIGURE 1:**
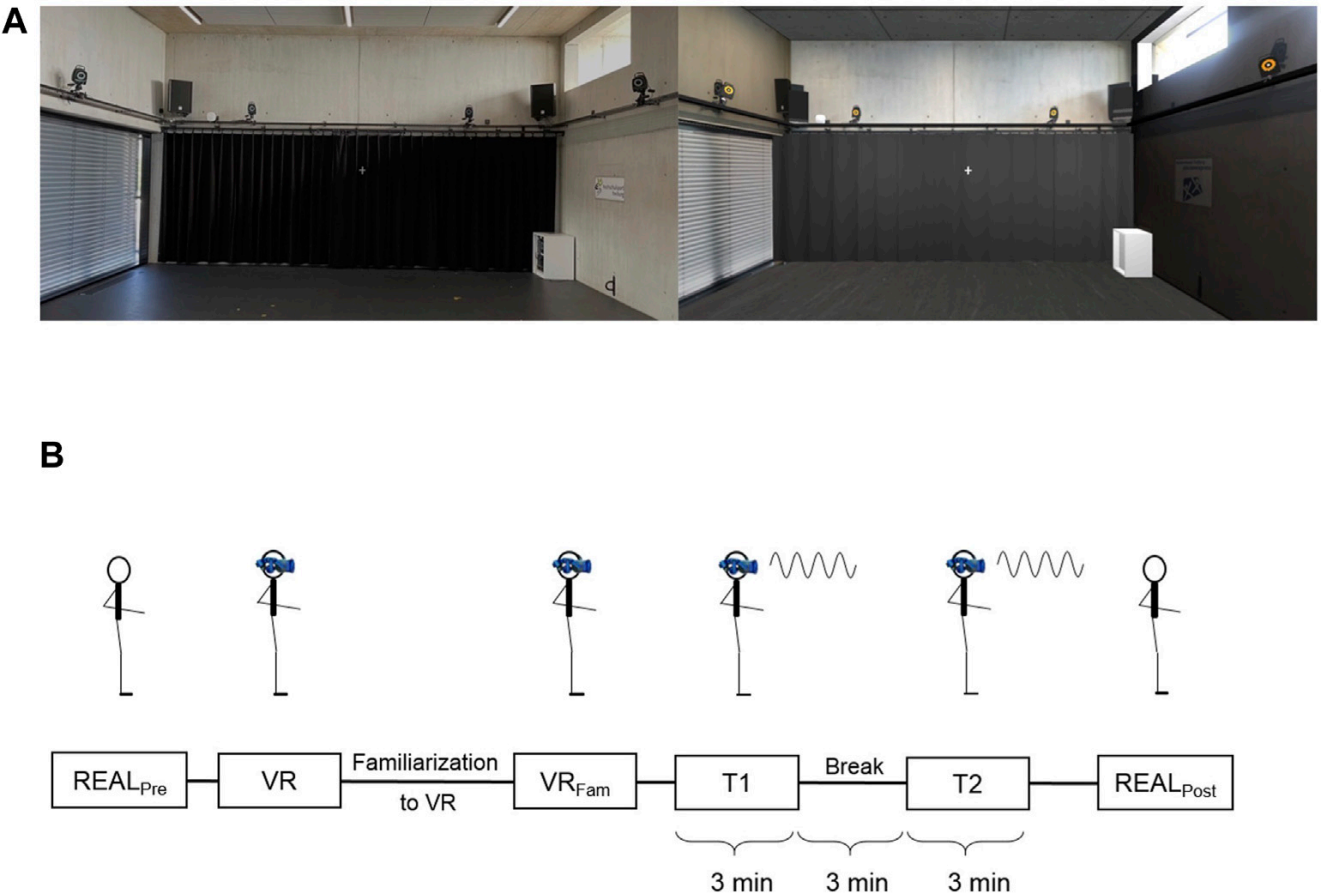
Experimental setup **(A)** Real environment (left) and virtual replica of the real environment (right) that was provided to the subjects via HMD **(B)** Schematic display of the experimental protocol.

The virtual environment was provided via a head-mounted display (HMD) (HTC Vive pro eye, HTC Corporation, Taoyuan City, Taiwan), with a resolution of 1440 × 1600 pixels per eye, a field of view of 110° and an update rate of 90 Hz. The stereo graphics were rendered with an AMD Ryzen 9 3900X processor and Nvidia GeForce RTX 2080 graphics card. The HTC Vive system includes a lighthouse tracking system, which tracks head position and orientation. This data updated the perspective displayed in the HMD, enabling the participants to move freely in the VR. The visual content in the HMD was a synthetic replicate of the real environment, thus a virtual measurement laboratory. The size of the virtual laboratory was scaled to match the real environment. The VR space was rendered in Unity3d (Unity Technologies, San Francisco, CA). Figure 1A illustrates a comparison of the real environment and our virtual replicate.

### Experimental Procedure

Initially, subjects were informed about the protocol and the measurement conditions. For each measurement condition, the participants were instructed to take a comfortable bipedal stance with feet shoulder-width apart and to keep arms to the side. During data acquisition, participants were asked to stand still, in a relaxed manner and to look at a “+” placed on the wall in front of them at eye level (in VR and real environment).

Figure 1B summarizes our experimental protocol. First, participant’s balance was assessed in the real environment with eyes open (“REAL_Pre_”) and in the virtual environment. In the virtual environment (“VR”), we measured balance immediately after the participant put on the HMD and after a 3-min familiarization phase in which the subject could move freely in the virtual space (“VR_Fam_”). Here optic flow provided by the HMD was not manipulated and provided reliable visual perception. Each of these conditions included three trials with each trial lasting 20 s.

In the following perturbation session, participants completed two 3-min trials (T1 and T2) while being exposed to continuous anterior-posterior rotation of the VR in the sagittal plane (pitch of the virtual room). The rotation was prescribed as a sinusoidal signal with an amplitude of 8°, a frequency of 0.2 Hz and no phase shift. These specifications were chosen as high amplitudes are known to evoke larger postural responses (Dokka et al., 2009; Chiarovano et al., 2015) and frequencies of 0.2 Hz were shown to be within a comfortable range with maximum entrainment in healthy adults (Peterka, 2002). The rotation axis of the visual field was 8.8 cm above the floor, thus approximately through the ankle joint axis. Trial length was chosen according to Amiri et al. (2019) who reported 3 min to be optimal for perturbation design, as it generally does not cause fatigue provided that adequate rest is allowed between trials. In-between both trials a 3-min break was implemented, where the subjects were asked to take a seat while still being immersed in the VR. After the break, a second 3-min perturbation trial (T2) was made. Subsequent to T2, subjects removed the HMD and balance was assessed in the real environment three times à 20 s (“REAL_Post_”).

### Measurements

Kinematic data were captured by a Vicon MX digital optical motion capture system with nine infrared cameras (Vicon Motion Systems Ltd., Oxford, United Kingdom) operating at 200 Hz. Thirty-nine retroreflective markers were attached to the subjects according to the Vicon Plug-in Gait Model. In combination with anthropometric measurements, this model allows to compute the three-dimensional coordinates of the joint centers, the segments’ center of mass as well as the whole body’s center of mass (COM).

### Data Analysis

Recorded data was analyzed using Matlab (The Mathworks, Natick, United States). Kinematic data were filtered using a fourth order low-pass Butterworth filter with a cutoff frequency of 8 Hz. For all trials, the filtered trajectory of the COM was used to calculate the mean speed of COM sway.

To compare COM sway during the perturbation trials to the non-perturbation trials, i.e. natural standing trials, data were segmented into nine blocks; each including four perturbation cycles (= 20 s as in the conditions preceding the perturbations). Mean COM sway speed was calculated for each block to show the temporal course over the exposure to perturbations.

Furthermore, the amplitudes of the anterior-posterior translations of the COM were converted into angles describing the rotation of the COM around the ankle joint. This conversion allows relating the amplitude of rotation angles to the amplitude of the visual stimulus. Herein, the so-called gain represents the ratio of COM response amplitude at the vision stimulus frequency (0.2 Hz) to the vision amplitude (8°) (Peterka, 2002). The magnitude of the response provides information about the relative weight of the visual contribution to balance and therefore enables a description of the dynamic characteristics of the balance control system. Hereby, a change in gain is interpreted as reweighting of the visual modality, that is, a decrease in gain indicates lower weighting (decreased coupling) to the visual stimulus. The frequency response function (FRF) at the stimulus frequency was obtained by dividing the discrete Fourier transform (DFT) of the time series of COM rotatory displacement and of the oscillatory component of stimulus motion. Ultimately, gain is the absolute value of the FRF at the stimulus frequency. As described in Jeka et al. (2010), we followed the standard cycle-by-cycle analysis, whereby the FRF of each cycle (in our case, 36 cycles with 5 s each) is processed separately. Gain values were also grouped in nine blocks and for each block the mean gain was calculated as the average of the DFT coefficients over the cycles.

### Statistics

We evaluated the dependent variables COM mean sway speed and gain. To test for the effect of the VR itself on postural stability, we first used a one-way repeated measure analysis of variance (rmANOVA) to compare between the visual conditions REAL_Pre_, VR and VR_Fam_.

To test for effects of optic flow perturbation, we conducted a rmANOVA on the average of each outcome measure taken at the time of interest: VR_Fam_, the mean of the first block (“First_T1” and “First_T2”) and the mean of the last block (“Last_T1” and “Last_T2”) of both trials, respectively, and the Post condition (REAL_Post_). Given a significant main effect of time, we performed the following planned, Šídák’s corrected post-hoc, pairwise comparisons: VR_Fam_ versus First_T1/First_T2, VR_Fam_ versus Last_T1/Last_T2, First_T1 versus Last_T1, First_T2 versus Last_T2, and VR_Fam_ versus REAL_Post_. To test for differences in visual afferent integration between First and Last block of T1 and T2, we used a rmANOVA of the gain values. We report effect size using partial eta squared (η_p_^2^) for main effects.

All statistical analyses were conducted using Prism 9 (GraphPad Software, San Diego, CA). All data are presented as mean value and 95% confidence intervals. For all statistical tests, the level of significance was set to *p* = .05.

## Results

**FIGURE 2:**
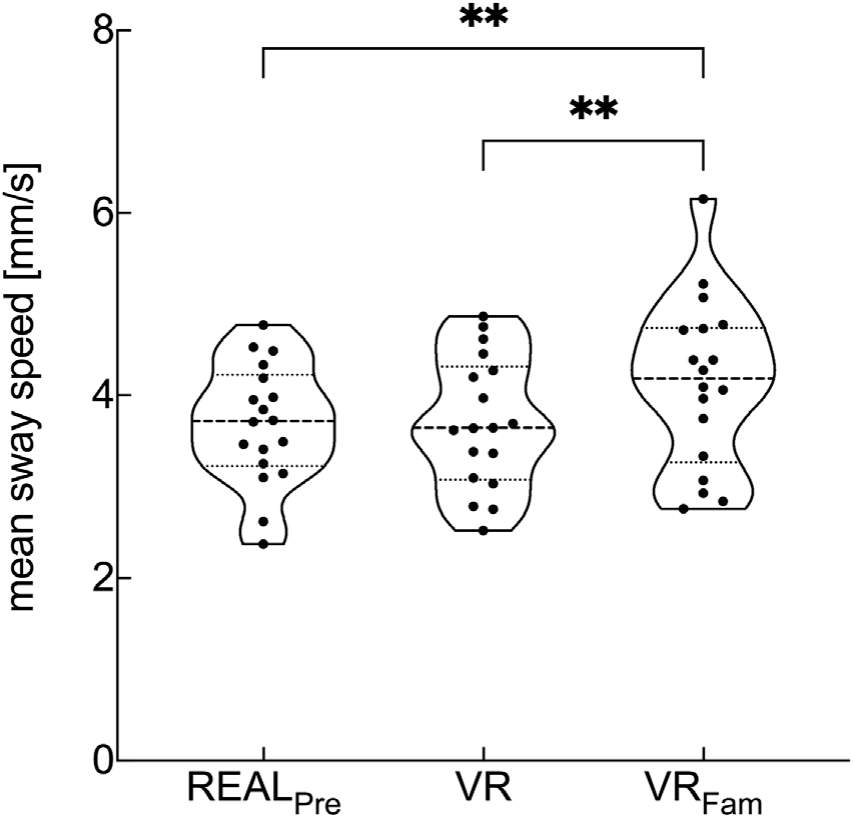
Violin plots of mean COM sway speed for conditions with reliable visual input preceding the optic flow perturbations. Visual conditions are real environment (REAL_Pre_), virtual replica of the real environment (VR), and virtual replica of the real environment after 3-min familiarization to VR (VR_Fam_). Violin plots represent data from each subject and show median values (dashed horizontal line), lower and upper 25th and 75th percentile values (dotted lines) and error bars (spanning smallest to largest individual values). **p < .01.

The visual condition showed a statistical main effect on the COM mean sway speed (*F*_1.75, 31.5_ = 12.27, *p* < .001, η_p_^2^ = .405). We found no significant difference in mean sway speed between REAL_Pre_ and VR. However, there was a significant difference between REAL_Pre_ and VR_Fam_ (*p* = .003) as well as between VR and VR_Fam_ (*p* = .002), respectively (Figure 2).

**FIGURE 3:**
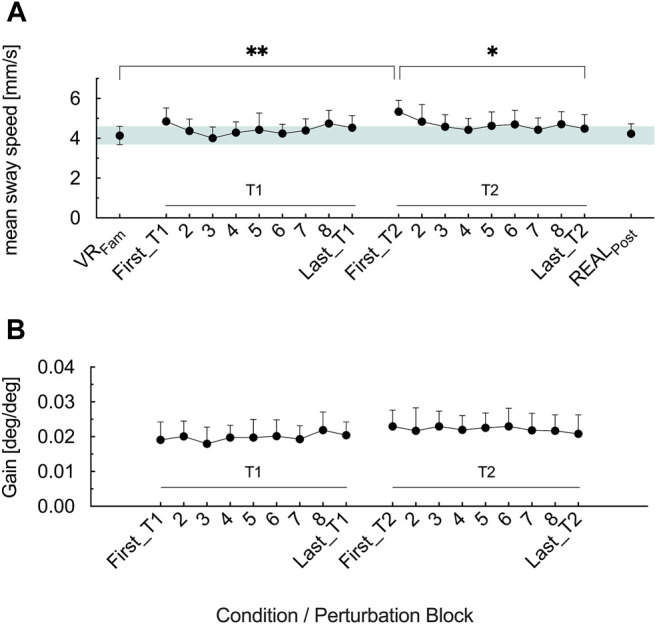
**(A)** Mean COM sway speed for the reference condition (VR_Fam_) and the nine perturbation blocks in T1 and T2 **(B)** Gain values for the nine perturbation blocks in T1 and T2. Error bars are the 95% CI of the mean. Green shaded horizontal bar in **(A)** highlights the 95% CI of VR_Fam_. *p < .05, **p < .01.

Figure 3A shows the effects of prolonged optic flow perturbations on postural stability (*F*_3.606, 61.3_ = 5.08, *p* = .002, η_p_^2^ = .23). In First_T1, optic flow perturbation elicited no greater mean sway speed (*p* = .238) compared to VR_Fam_, whereas First_T2 showed greater mean sway speed as in VR_Fam_ (*p* = .001). From First_T1 to Last_T1, mean sway speed did not change (*p* = .963). In the second trial, mean sway speed decreased from First_T2 to Last_T2 (*p* = .014) and did not show differences to VR_Fam_ anymore (*p* = .856). No difference existed in the COM mean sway speed of First_T1 and First_T2 (*p* = .686). Immediately following cessation of optic flow perturbations of the second trial (POST_EO_), participants exhibited similar (*p* = .999) mean sway speed as during VR_Fam_.

For the gain values we found no main effect of time (*F*_2.362, 40.15_ = 5.08, *p* = .496, η_p_^2^ = .043) (Figure 3B).

To verify whether significances were merely masked by block building (nine blocks à four perturbation cycles) especially at the beginning of representing the optic flow stimulus, we subsequently “zoomed in” the first block of T1 and T2 (= first four perturbation cycles) and checked for perturbation-to-perturbation differences. Therefore, we conducted a rmANOVA for both First_T1 and First_T2 and compared it with the reference condition VR_Fam_.

**FIGURE 4:**
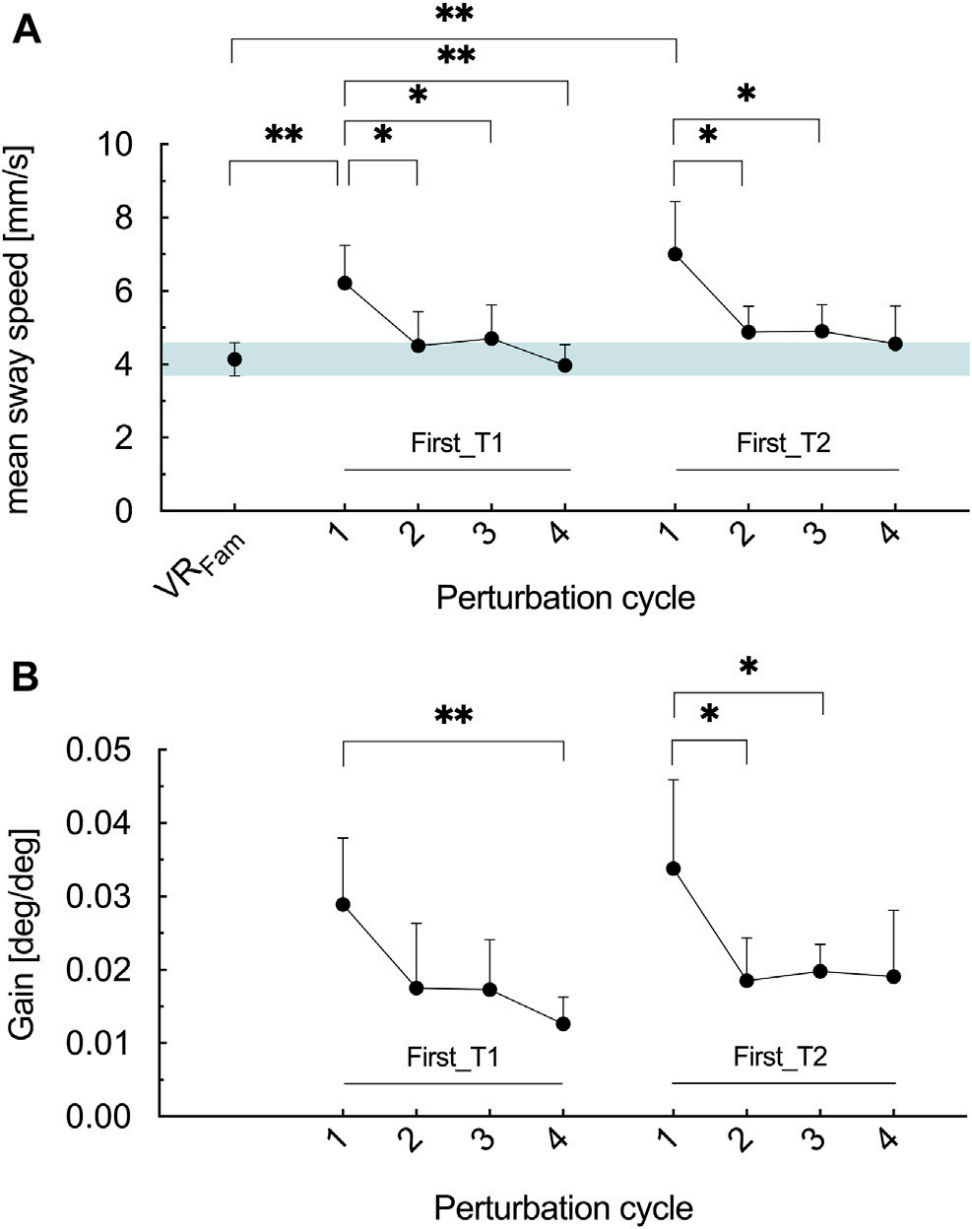
**(A)** Mean COM sway speed for the reference condition (VR_Fam_) and the four perturbation cycles of block one in T1 (First_T1) and T2 (First_T2) **(B)** Gain values for the four perturbation cycles of block one in T1 (First_T1) and T2 (First_T2). Error bars are the 95% CI of the mean. Green shaded horizontal bar in **(A)** highlights the 95% CI of VR_Fam_. *p < .05, **p < .01.

Across perturbation cycles, mean sway speed exhibited significant main effects in First_T1 and First_T2 (*F*_2.646, 44.98_ = 8.826, *p* < .001, η_p_^2^ = .342 and *F*_2.087, 35.48_ = 7.168, *p* = .002, η_p_^2^ = .297, respectively) (Figure 4A). Results revealed that the first cycle of optic flow perturbation elicited 50% greater sway speed in the T1 (*p* = .003) and 69% in T2 (*p* = .003), respectively, compared to VR_Fam_. In T1, the first perturbation cycle elicited significantly greater mean sway speed than the second (*p* = .025), the third (*p* = .017) and the fourth (*p* = .001) perturbation cycle. In T2, mean sway speed during the first perturbation cycle was greater than during the second (*p* = .022) and the third perturbation cycle (*p* = .036). There tended to be a difference between the first and the fourth perturbation cycle (*p* = .051).

Similarly, as for mean sway speed, time exhibited a significant main effect on the gain values in First_T1 and First_T2 (*F*_1.848, 31.41_ = 5.548, *p* = .01, η_p_^2^ = .246 and *F*_1.950, 33.14_ = 3.691, *p* = .037, η_p_^2^ = .178, respectively) (Figure 4B). In T1, gain values in the first perturbation cycle were similar as in the second (*p* = .154) and the third (*p* = .069) but greater than in the fourth perturbation cycle (*p* = .006). In T2, greater gain values were measured for the first perturbation cycle compared to the second (*p* = .045) and the third (*p* = .038) perturbation cycle. The fourth cycle did not differ to the first (*p* = .16).

## Discussion

The main purpose of this study was to investigate the effect of continuous optic flow perturbations as a trigger of sensory conflict on postural stability. We employed a swinging room paradigm where a scaled and lifelike VR rotated sinusoidally around the subjects’ ankle joint in the sagittal plane to induce the visual perception of self-motion that contradicts vestibular and somatosensory sensory input. By this means, we aimed to elicit sensory re-weighting processes that could be used for balance training purposes.

The results suggest that these perturbations produce impaired balance control compared to quiet standing with reliable visual information, but only in a specific time domain. The main findings of this study where that 1) only the first perturbation cycle created sensory conflicts that were strong enough to elicit postural instability and 2) visual afferents were downregulated after the first perturbation cycle to reduce the sensory conflict and therefore postural instability.

VR-induced optic flow perturbation is thought to be one mean for balance training, as the motor system increases robustness of motor control in the presence of perturbations (Santuz et al., 2018; Munoz-Martel et al., 2019). Training programmes using sensory perturbations to exercise dynamic stability can enhance sensory information processing within the motor system (Hamed et al., 2018). To trigger the additional response and thus have a training effect, however, the perturbations must be challenging enough (Hamed et al., 2018). Chiarovano et al. (2015) and Heidner et al. (2020) demonstrated that 25 or 30 s exposure to optic flow perturbations in VR can cause postural instability. The authors also suggest a clinical application of these perturbations as their findings demonstrate that motor control strategies can be challenged by optic flow perturbations without physically perturbing the subject (Heidner et al., 2020). Yet, past investigations provided rational that balance control in VR is per se compromised with respect to the real environment (Horlings et al., 2009; Kelly et al., 2019). Therefore, one could argue that postural instability induced by moving room paradigms is not solely due to the optic flow perturbations, but also a consequence of the VR itself. In our study, however, we found no difference in body sway between REAL_Pre_ and VR indicating that our VR itself did not trigger any postural instability. Our results support the work by Assländer and Streuber (2020), showing that a state-of-the-art VR device with photorealistic and lifelike VR scenarios provides visual conditions that equal real environments and consequently evoke body sway behaviour that is similar to real life. Menzies et al. (2016) also support the notion that better technical devices reduce spontaneous sway and dedicated their findings to higher fidelity of the visual surround. However, we observed greater postural instability after the 3-min familiarization (VR_Fam_) compared to the initial VR condition and REAL_Pre_. Work by Robert et al. (2016), who also used a recent VR device, might explain this finding. For their VR content, the authors used a 3D filmed visual representation that showed the laboratory room. They reported no difference for VR and the real environment for static balance. However, dynamic balance tasks were more perturbed in the VR compared to the real environment. They conclude that dynamic or more challenging balance tasks are impaired in VR because of sensory conflicts due to for example latency of the HMD. The 3-min walking familiarization in our study might have triggered this phenomenon as well. Consequently, we referenced postural stability during the optic flow perturbations to VR_Fam_, to ensure that increased instability during the perturbations is a consequence of the perturbation itself.

This postural instability during the perturbation trials was solely observed for First_T2 compared to VR_Fam_; for First_T1 mean sway speed was not increased compared to VR_Fam_. In an experiment with support-surface perturbations, Horak et al. (1989a) observed overreacting postural responses when a small platform perturbation was preceded by a series of larger perturbations. This phenomenon was also shown for optic flow perturbations (O’Connor et al., 2008). Similarly, in our study, subjects may have shown an overreacting postural response in First_T2 because they were expecting a larger optic flow stimulus. Mean sway speed then adapts, with Last_T2 resembling VR_Fam_.

Besides this, the only difference between T1 and T2 is of a temporal nature. Although speculative, the additional time spent in the VR may have increased visual reliance on the virtual scenery, thus becoming more prone to optic flow perturbations in T2 than in T1. The 3-min static break between T1 and T2 may have further facilitated this effect. The fact that the optic flow perturbation did not induce significant postural instability in First_T1 contrasts with the literature (Chiarovano et al., 2015; Heidner et al., 2020). To account for this incoherent and unexpected finding, we more closely inspected the first block in T1 and T2, respectively, and conducted a perturbation-to-perturbation analysis. Striking here is that the first perturbation cycle of each trial elicited greater mean sway speed compared to VR_Fam_ and that mean sway speed during the first perturbation cycle was also greater than during the remaining cycles of the first blocks in T1 and T2, respectively. This suggests that our optic flow perturbation paradigm can only initially trigger a sensory conflict, which is sufficient to cause postural instability. This finding is supported by Nashner and Berthoz (1978) and Bronstein (1986), who also showed this rapid habituation of sway response to visual scene movement. Especially the first presentation of optic flow perturbation induced larger instability compared to the following presentations (Bronstein, 1986). In a protocol with 45 s exposure to sinusoidal optic flow perturbations O’Connor et al. (2008) observed substantial reduction in sway speed in the first 10 s. The authors suggest changes in sensory reweighting as a possible mechanism. That subjects have the greatest reduction after first trial was also demonstrated by Sundermier et al. (1996) who exposed subjects to successive forward translations of the visual field. This observation may have been induced by the unexpected incongruence of vision with other balance-relevant inputs that is destabilizing (Sundermier et al., 1996). If optic flow perturbations are continuous and expectable, which is also true for our sinusoidal application, it is plausible that subjects were able to anticipate the perturbation after the first exposure and subsequently resisted to instability. This is in line with the findings of Chander et al. (2019) or Guerraz et al. (2001), who showed that expected optic flow perturbations resulted in smaller effects on postural control compared to unexpected optic flow perturbations. Moreover, even with pseudorandom and therefore unpredictable stimuli, the results by Peterka (2002) did not show evidence of adaption or habituation in the sway response after the first perturbation cycle. In our study, the continuous optic flow perturbations may have caused the subjects to adapt using a predictive strategy (Thompson and Franz, 2017) and thereby exhibit anticipatory behaviour (Amiri et al., 2019) that resists balance perturbation. In contrast, the first perturbation cycle in each trial differed by its abrupt start and the associated discrete characteristic from the following continuous cycles. The abrupt start results from the characteristic of sinusoidal stimuli. In contrast to a cosine or smoothstep function, a sine (with no phase shift) has its peak in the speed profile directly at stimulation onset. Abrupt optic flow perturbations are unpredictable and require a greater reactive and compensatory response that is more reflective of the direct effect of the optic flow perturbation (Kajrolkar et al., 2014).

This impact of the first perturbation cycle can be explained by the feedback model-based interpretation of balance control where feedback is provided by a weighted combination of sensory inputs, which are dynamically adjusted to maintain stability after changes in environmental conditions (Peterka, 2002). This has functional consequences for postural stability. In situations where environmental conditions suddenly change, i.e. perturbation onset, the availability of sensory orientation cues is altered, which causes a transient period of either under- or over-generation of corrective torque due to inappropriate (slow) adjustments of sensory weights for the new environmental condition (Peterka and Loughlin, 2004). Ultimately, the ability to quickly react to sudden changes is an important function of the balance control mechanism, as sudden environmental changes initiate dynamic postural adjustments that are centrally mediated and thus cannot occur instantaneously (Peterka, 2018). This poses a threat to stability (Polastri et al., 2012) and leaves subjects vulnerable to transient instability. Deficits in sensory reweighting are therefore considered one of the most critical factors for balance control in various patient groups and elderly (Horak et al., 1989b).

In the existing literature, these transient responses are commonly masked by whole-trial analyses (Reed et al., 2020) or the first perturbation cycle is discarded to avoid transient responses in order to get more reliable results for describing steady-state balance as a function of sensory input (Carpenter et al, 2001). However, in the perturbation-to-perturbation analysis, the transient instability is prominent and relatable to the enhanced integration of the visual afferents into the postural system as revealed by the increased gain values for the first perturbation cycle. The optic flow perturbation therefore has greater impact for postural control. Consequently, the VR-induced optic flow perturbation provides an initial destabilizing period preceding more stable postural control highlighting the role played by the dynamic regulation of sensorimotor integration. One interpretation of our findings is that the adaptation within trials may be explained by the decreased gain values and therefore the relative downregulation of visual feedback to reduce instability in the system for the following perturbation cycles. Reciprocally, subjects might have increased their awareness of reliable, available sensory information (Jeka et al., 2010). This can have implications for the rehabilitation of subjects with strong visual dependency for balance control, for example fall-prone elderly (Lord and Webster, 1990; Jeka et al., 2010). Incorporating optic flow perturbations during rehabilitation exercises may reweight sensory neural processing towards an upregulation of proprioceptive or vestibular inputs and reduce the dependency on vision to guide postural control.

Based on EEG data from Peterson et al. (2018) the time-dependent changes we report here may be simultaneously accompanied by changes in cortical activation. The authors used transient optic flow perturbations in young adults walking on a balance beam. They report increased electrocortical activation in parietal, occipital, and cingulate areas due to conflicting sensory information during balance. This suggests that such perturbations promote motor learning of a balance task in brain areas associated with integrating visual information and may thus reflect the brain’s ability to adapt to variations in sensory input (Peterson et al., 2018).

### Limitations

We observed no difference in postural stability between reality and VR. After the dynamic familiarization period, however, subjects standing balance was decreased. Due to lacking a control group who spent the familiarization statically, it remains questionable whether this increase in postural instability is a result of the walking interaction or an aspect of time or visual fatigue. To account for this, we referenced the perturbation trials to VR_Fam_ to ensure that postural instability is a consequence of the optic flow perturbation and not of the VR itself. After the first perturbation cycle, postural instability is no longer induced. Thus, it is not clear, whether the optic flow perturbations paradigm used in this study was not challenging enough for the participants. However, numerous studies have shown evidence for sway responses to sinusoidally optic flow perturbations, even after repeated exposure (van Asten et al., 1988; Peterka and Benolken, 1995). Another important limitation of our study design is that the results do not allow separating whether the decrease of postural instability after the first perturbation cycle is due to the continuous or the predictable nature of the stimulus. This hampers the development of optimal metrics for VR based rehabilitation paradigms.

### Perspective

Our findings come with potential implications within the area of VR-based training and rehabilitation of balance. Many older adults may fall not because they are too weak or too stiff to respond, but as the results by Peterka and Loughlin (2004) predict, their risk of falling increases when environmental conditions change due to the too slow regulation of sensory weights. This is leveraged by the fact that, for instance, elderly succumb to a loss of reliability of the sensory feedback and rely more on vision than somatosensory and vestibular systems to maintain their balance (Lord and Webster, 1990; Jeka et al., 2010). This inaccurate perception may lead to compensatory responses that are inappropriate to correct for the loss of stability (Anson and Jeka, 2010), because somatosensation is the most important system for postural control, as it provides the fastest information processing (Campbell, 2007). Against this background, optic flow perturbations might be a helpful tool that should be considered when developing rehabilitation programs, as they could help patients to decrease reliance on visual information during balance control and upregulate reliance on somatosensory information for motor programming (Lee et al., 2021; Han et al., 2022). The implementation of this novel approach may enhance the activity of the somatosensory pathways to the postural system due to limited visual information input (Kim et al., 2021). Future research is needed to investigate the effects of training programs that include optic flow perturbations on postural control in individuals with altered somatosensory input due to musculoskeletal injury or aging. Furthermore, VR research and rehabilitation lacks of perturbation paradigms for creating effective and repeatable sensory conflicts. These sensory conflicts are needed to induce sensory reweighting and to improve the dynamic regulation of sensorimotor integration. Amiri et al. (2019) recently suggested using transient optic flow perturbations in random directions, as these stimuli are unpredictable and abrupt. Empirical evidence for this suggestion needs to be established yet.

## Conclusion

Continuous sinusoidal optic flow perturbations appear not to be suitable to provide persisting sensory conflicts and hence to challenge the postural system sustainably. Therefore, it seems questionable to use these predictable perturbation paradigms as a tool for balance training. However, particularly the first perturbation cycle with its discrete characteristic is suitable for triggering instability. The application of discrete perturbations may elicit separate, distinct corrections that may be less easy to adapt to. Consequently, this sort of optic flow perturbation appears to be promising for balance training and balance rehabilitation.

## Data Availability

The raw data supporting the conclusions of this article will be made available by the authors, without undue reservation.
